# erbB3 recruitment of insulin receptor substrate 1 modulates insulin-like growth factor receptor signalling in oestrogen receptor-positive breast cancer cell lines

**DOI:** 10.1186/bcr3018

**Published:** 2011-09-22

**Authors:** Janice M Knowlden, Julia MW Gee, Denise Barrow, John F Robertson, Ian O Ellis, Robert I Nicholson, Iain R Hutcheson

**Affiliations:** 1 Welsh School of Pharmacy, Cardiff University, Redwood Building, King Edward VII Avenue, Cardiff, CF10 3NB, UK; 2 Professorial Unit of Surgery, Nottingham City Hospital, Hucknall Road, Nottingham, NG5 1PB, UK; 3 Department of Histopathology, Nottingham City Hospital, Hucknall Road, Nottingham, NG5 1PB, UK; 4 Department of Pharmacology, Radiology & Oncology, Cardiff University, School of Medicine, Heath Park, Cardiff, CF14 4XN, UK

**Keywords:** breast cancer, erbB3, IRS-1, IGF-IR, resistance

## Abstract

**Introduction:**

Recently we reported that insulin receptor substrate 1 (IRS-1), classically an adaptor protein for the insulin-like growth factor type I receptor (IGF-IR), associates with the epidermal growth factor receptor in oestrogen receptor (ER)-positive (ER+) tamoxifen-resistant breast cancer cells. In this study, we examined whether IRS-1 also associates with another erbB receptor family member, erbB3, and what impact this might have on IGF-IR signalling in three ER+ breast cancer cell lines.

**Methods:**

Immunoprecipitation and Western blot analysis were utilised to examine the potential association between erbB3 and IRS-1 in MCF-7, T47D and BT-474 cells in the absence and presence of the erbB3/4 ligand heregulin β1 (HRGβ1). Subsequently, the impact of a selective IGF-IR/IR inhibitor 4-anilino-5-bromo-2-[4-(2-hydroxy-3-(*N*, *N*-dimethylamino)propoxy)anilino]pyrimidine on this association and HRGβ1 signalling was assessed in these cell lines. Immunohistochemical analysis of a small cohort of ER+ breast cancer patient samples was also performed to determine the potential clinical relevance of this novel interaction.

**Results:**

Immunoprecipitation and Western blot analysis revealed an interaction between erbB3 and IRS-1 in MCF-7, T47D and BT-474 cells, with HRGβ1 significantly enhancing this recruitment and promoting IRS-1 phosphorylation at Y612. IRS-1 participates in erbB3 signalling in MCF-7 and T47D cells as IRS-1 knockdown impaired HRGβ1 signalling. Importantly, recruitment of IRS-1 by erbB3 reduced IRS-1 association with IGF-IR in MCF-7 and T47D cells, whilst blockade of IGF-IR-enhanced erbB3-IRS-1 interaction and sensitised both cell lines to HRGβ1, allowing HRGβ1 to override IGF-IR blockade. Consequently, suppression of IRS-1 signalling enhanced the effects of IGF-IR inhibition in these cells. This novel interaction may have clinical relevance, as immunohistochemical analysis of a small ER+ breast tumour series revealed significant positive correlations between phosphorylated IRS-1 Y612 expression and total erbB3, phosphorylated Akt and Ki-67 expression.

**Conclusions:**

IRS-1 can be recruited to IGF-IR and erbB3 in ER+ breast cancer cells, and this provides an adaptive resistance mechanism when these receptors are targeted individually. Consequently, cotargeting IGF-IR and either erbB3 or IRS-1 should prove to be a more effective strategy for the treatment of ER+ breast cancer.

## Introduction

There is strong experimental and clinical evidence implicating the insulin-like growth factor type I receptor (IGF-IR) in breast cancer development and growth [1-3]. The IGF-IR, which belongs to a family of receptor tyrosine kinases that includes the insulin receptor (IR), has been found to be expressed in a high percentage of breast tumours, where its expression is positively correlated with oestrogen receptor (ER) status and is usually coexpressed with markers of a better overall prognosis [2,4-6]. Expression of the IGF-IR has also been demonstrated in the majority of ER+ breast cancer cell lines [7,8]. Indeed, in MCF-7 cells, IGF-IR has been shown not only to be a key receptor in mediating hormone-sensitive growth but also to engage in significant cross-talk with ER [9,10]. Importantly, this leads to synergistic interactions between ER and IGF-IR signalling to promote efficient growth responses [2].

However, conversely, increased expression and activation of IGF-IR and its associated downstream signalling components have also been reported in some clinical breast cancers and have been linked to disease progression and recurrence [11,12]. On the basis of these data, IGF-IR has been identified as a potential therapeutic target for the treatment of breast cancer [13]. Activation of the IGF-IR promotes binding of insulin receptor substrate (IRS) members, a family of structurally related adaptor molecules which have classically been identified as key signalling intermediates of the IR and IGF-IR [14]. Binding results in phosphorylation of their carboxyl termini at multiple tyrosine residues, and these phosphotyrosine residues provide docking sites for the recruitment of key signalling pathways, such as the mitogen-activated protein kinase (MAPK)/extracellular signal-regulated kinase 1/2 (ERK1/2) and phosphatidylinositol 3-kinase (PI3K) pathways [15]. These signalling cascades can mediate mechanisms underlying tumour growth and progression, implicating a potential role for IRS members in oncogenesis [1,15-18]. Indeed, IRS-1 has been reported to be overexpressed and constitutively phosphorylated in breast tumours [18,19], and high expression of this adaptor protein has been associated with lymph node metastases and poor patient prognosis [11,20,21]. Furthermore, IRS-1 and IRS-2 have been implicated in the regulation of proliferation, survival and metastatic potential in a range of breast cancer cell lines [17].

However, there is now increasing evidence that IRS-1 is not restricted to binding to IR/IGF-IR but is also capable of associating with a variety of other signalling-related proteins [17]. One such protein is the epidermal growth factor receptor (EGFR), a member of the erbB receptor tyrosine kinase family also comprising erbB2, erbB3 and erbB4 and which has been shown to play a central role in driving both *de novo *and acquired anti-hormone-resistant growth and invasion in breast cancer [22-25]. Evidence of an EGFR-IRS-1 interaction arises from reports by Fujioka and colleagues [26,27], who reported that the phosphorylated NPXY motifs in activated IR and IGF-IR to which IRS molecules bind are also present in the C-terminus region of activated EGFR and were indispensable for EGF-induced IRS-1 tyrosine phosphorylation in EGFR-transfected CHO cells [27]. Furthermore, a potential interaction between EGFR and IRS-1 has been predicted on the basis of the binding of peptides, representing the physical sites of EGFR tyrosine phosphorylation, to protein microarrays comprising all Src homology 2 and phosphotyrosine binding domains encoded in the human genome [28]. Recently, we provided strong evidence that IRS-1 can function as a key signalling intermediate for EGFR, a receptor that drives the growth of a tamoxifen-resistant MCF-7 breast cancer cell line [29]. In these cells, we showed that IRS-1 physically complexes with EGFR and is preferentially phosphorylated on Y896, a Grb2-binding/MAPK recruitment site [15]. Moreover, EGFR was the dominant recruiter of IRS-1, which thus served to limit the availability of IRS-1 to associate with IGF-IR in these cells and, as a result, suppressed IGF-IR signalling via this receptor.

Other erbB receptors are also prevalent in breast cancer, and their interplay with IRS-1 remains unknown. Of note, we have previously shown [30] that erbB3 is commonly expressed in clinical breast tumours alongside one of its ligands, heregulin β1 (HRGβ1). Interestingly, erbB3 also possesses the NPXY motifs recognized by IRS proteins [31] and as such may bind IRS-1 in breast cancer cells. Indeed, such an association has again been predicted on the basis of protein microarray studies [28]. A role for erbB3 in breast cancer has only recently become appreciated [32,33], with overexpression of erbB3 shown to be positively associated with metastasis [34], increased histological grade [35] and tumour recurrence [36]. There is also growing awareness of the importance of the erbB2/erbB3 heterodimer in breast cancer progression. Heterodimers between these two receptors have been shown to form the most potent mitogenic and transforming receptor complex *in vitro *[37], and coexpression of erbB2 and erbB3 have been shown to be significantly associated with decreased survival in breast cancer patients [38]. Interestingly, erbB3 signalling has also been implicated in mediating resistance to IGF-IR-targeted agents in hepatocellular carcinoma cells [39], but whether it plays a similar role in breast cancer remains to be determined. In the present study, using a panel of ER+ breast cancer cell lines, we examined for the first time whether IRS-1 can contribute to erbB3 signalling in breast cancer and what impact this may have on IGF-IR signalling. We show that IRS-1 is recruited to erbB3 following HRGβ1 treatment in these cells and demonstrate that this novel interaction can serve to reduce the association between IRS-1 and IGF-IR and inhibits signalling via this receptor. We show in turn that suppression of IGF-IR by the use of a tyrosine kinase inhibitor and siRNA technology can promote erbB3 downstream signalling by reinforcement of erbB3 interplay with IRS-1. This provides a potential novel resistance signal, which, when targeted, may generate more effective inhibition of cell growth compared to IGF-IR treatment alone.

## Materials and methods

### Cell culture

MCF-7 cells (a gift from AstraZeneca Pharmaceuticals, Cheshire, UK) and T47D cells (American Type Culture Collection (ATCC), Manassas, VA, USA), which are both nonamplified erbB2 breast cancer cell lines, were grown in RPMI medium containing 5% FCS and glutamine (4 mM). Both cell lines were maintained at 37°C in a humidified 5% CO_2 _atmosphere. BT-474 over amplified erbB2 breast cancer cells (ATCC) were grown in RPMI medium containing 10% FCS and glutamine (4 mM).

### Experimental procedures

The cell lines were grown to 70% confluence prior to transfer into phenol red/steroid-and serum growth factor-free dendritic cell conditioned medium (Biosynergy Europe, Cambridge, UK) for 24 hours followed by exposure for up to 20 minutes to either 0.1 to 10 ng/ml HRGβ1 or 10 ng/ml IGF-I in 10 mM acetic acid/0.1% BSA or appropriate vehicle control. To examine the effects of pharmacological blockade of IGF-IR, cells were incubated in phenol red-free (white) RPMI medium supplemented with 5% FCS and either the IGF-IR/IR tyrosine kinase inhibitor 4-anilino-5-bromo-2-[4-(2-hydroxy-3-(*N*, *N*-dimethylamino)propoxy)anilino]pyrimidine (ABDP) (1 μM in dimethyl sulphoxide, AstraZeneca, Macclesfield, UK) [40] or appropriate vehicle control for 1 to 2 days. All experiments were performed at least three times. Cells were then lysed to measure protein expression.

### siRNA studies

Dharmacon SMARTpool siRNA Design specific for IRS-1 (IRS si), erbB3 (3 si) or IGF-IR (IGF si; all 20 mM, Dharmacon RNAi Technologies, Lafayette, CO, USA) were mixed with DharmaFECT 1 transfection reagent (lipid; Dharmacon RNAi Technologies) at a ratio of 10 μl of siRNA to 1 μl of lipid and incubated at room temperature for 20 minutes. The mix was added to the cells, which were maintained in white RPMI medium containing 5% FCS to give a final siRNA concentration of 100 nM per dish. Control experiments consisted of transfection with the ON-TARGETplus Nontargeting siRNA control pool (100 nM; Dharmacon RNAi Technologies), medium only (nontransfected cells) or lipid. All experimental arms were set up in duplicate. Cells were incubated in growth medium containing either IRS si, 3 si, IGF si or control (C si) (100 nM for each) for 4 days prior to treatment with either 10 ng/ml HRGβ1 or vehicle alone for 5 minutes. To examine the effect of IRS-1 knockdown and IGF-IR blockade, cells were incubated in medium containing either 100 nM C si, 100 nM IRS si, 1 μM ABDP or a combination of these treatments prior to a 5-minute incubation with HRGβ1 (10 ng/ml). The cells were then lysed, and protein extracts were examined by Western blot analysis.

### Immunoprecipitation and Western blot analysis

Fresh cell lysates containing 500 μg of protein were immunoprecipitated using 1 μg of specific antibody as described previously [24]. Protein samples from either immunoprecipitation or total cell lysates (20 to 50 μg) were separated on a 7.5% polyacrylamide gel and then transblotted onto nitrocellulose membrane as described previously [24]. The antibodies used were directed against total EGFR (SC-03), total erbB2 (SC-284), total erbB3 (SC-285), total IGF-IR (SC-712), total IRS-1 (SC-7200; Insight Biotechnology Ltd, Wembley, UK), total and phosphorylated Akt YS473, ERK1/2 and phosphorylated c-erbB3 Y1289 (Cell Signaling Technology, Hitchin, UK), phosphorylated EGFR (Y1068), phosphorylated IRS-1 (Y612 and Y896; BioSource International, Camarillo, CA, USA), β-actin (Sigma-Aldrich, Dorset, UK) and specific phosphorylated IGF-IR Y1316 (a kind gift from AstraZeneca, Macclesfield, UK). The Western blots were then scanned by densitometry to provide data for semiquantification. Each experiment was performed at least three times with representative gels shown in figures.

### Cell proliferation studies

Cells were seeded at 40, 000 cells per well overnight in phenol red-free RPMI medium supplemented with 5% FCS and then incubated in fresh medium containing 0.1 to 1 μM ABDP, 10 ng/ml HRGβ1, vehicle control or a combination of these agents for 4 days. Cell population growth was evaluated by means of trypsin dispersion of the cell monolayers, and cell number was measured using a COULTER COUNTER (Beckman Coulter (UK) Ltd, High Wycombe, UK). All experiments were performed in triplicate.

### Clinical series

A small historical series of 50 primary tumours were excised from ER+ patients with histologically proven breast cancer who had presented for surgery at Nottingham City Hospital (Nottingham, UK) from 1984 to 1987. Representative tissue samples from these tumour samples were fixed routinely in 4% formal saline and embedded in paraffin. No patient had previously received any form of adjuvant endocrinological or cytotoxic therapy. The use of these samples for research purposes, without the requirement of further patient informed consent, was approved by Nottingham Research Ethics Committee 2 under the title 'Development of a molecular genetics classification of breast cancer' (C2020313).

### Immunocytochemical assays

Immunocytochemical assays for phosphorylated Akt, Ki-67 and total erbB3 and specific phosphorylated IGF-IR Y1316 were performed on 50 primary ER+ breast tumours as previously described [6,30,41] and the clinicopathological parameters for the clinical set of these tumours are as shown (Additional file 1, Table S1). For the detection of phosphorylated IRS-1 Y612, paraffin wax sections from each tumour sample were dewaxed using xylene treatment and then rehydrated through graded ethanols to PBS. Endogenous peroxidases were destroyed by immersing the sections in 3% hydrogen peroxide prepared in methanol for 5 minutes, followed by rinsing with distilled water for 5 minutes. Antigen retrieval was achieved by pressure cooking the slides in 0.01 M sodium citrate buffer, pH 6.0, for 4 minutes. Slides were then immersed in slowly running tap water for 10 minutes before being transferred to PBS for 5 minutes. Sections were blocked in 1% l BSA for 5 minutes prior to incubation overnight at 37°C in anti-phosphorylated IRS-1 Y612 rabbit primary antibody diluted 1:50 in PBS. Sections were washed for 3 minutes in PBS, washed twice for 5 minutes in 0.02% PBS-Tween 20 and then incubated for 2 hours at room temperature in a peroxidase-labelled polymer secondary antibody EnVision Kit (Dako Ltd, Ely, UK). Slides were then washed for 3 minutes in PBS, washed twice for 5 minutes in 0.02% PBS-Tween 20 and incubated for 10 minutes at room temperature in EnVision DAB chromogen solution (diaminobenzidine; Dako Ltd). Slides were then rinsed twice for 2 minutes in distilled water, incubated in 0.5% methyl green for 25 seconds as a counterstain, rinsed in distilled water and allowed to air-dry before DPX mountant (a mixture of distyrene, a plasticizer and xylene) was applied. Combined cytoplasmic and plasma membrane staining intensity and percentage positivity were assessed by HScore analysis as described previously [41]. Expression of phosphorylated membrane and cytoplasmic IRS-1 Y612 has previously been verified by immunocytochemistry in ER+ breast cancer cell lines [29].

### Statistics

For immunocytochemical analysis of clinical material, Hscores were compared using Spearman's rank-correlation and Mann-Whitney *U *tests for nonparametric data. For experimental growth studies, overall differences between the control and treatment groups were determined by analysis of variance with *post hoc t*-tests with the Bonferroni adjustment factor. A two-sided *t*-test was performed on the densitometry values obtained following Western blot analysis. Differences were considered significant at the P ≤ 0.05 level.

## Results

### Heregulin β1 promotes phosphorylation of insulin receptor substrate 1 in MCF-7, T47D and BT-474 oestrogen receptor-positive breast cancer cells

**Figure 1:**
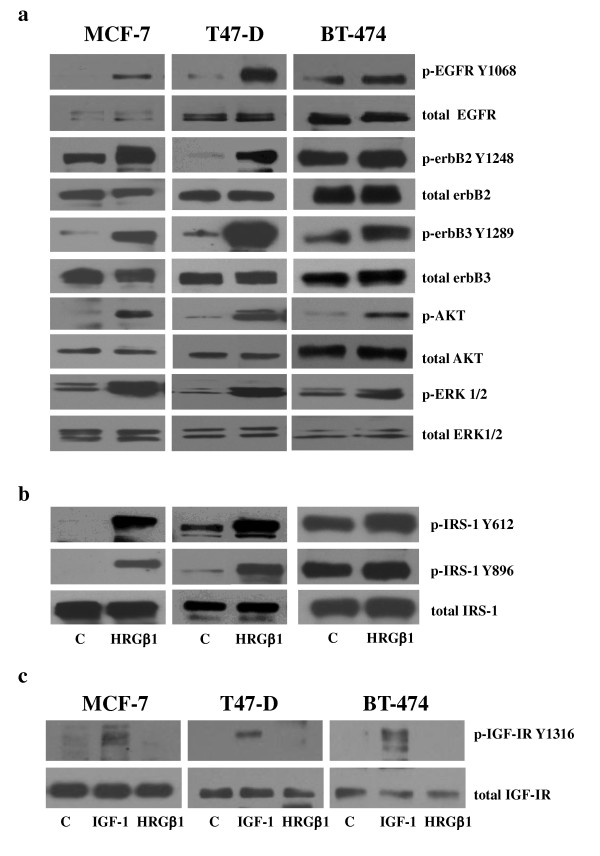
**Western blot analysis**. **(a) **Phosphorylated and total epidermal growth factor receptor (EGFR), erbB2, erbB3, Akt and extracellular-signal regulated kinase 1/2 (ERK1/2) and **(b) **phosphorylated and total insulin receptor substrate 1 (IRS-1) protein expression following treatment of MCF-7, T47D and BT-474 breast cancer cells with either heregulin β1 (HRGβ1) (10 ng/ml) or vehicle control for 5 minutes. **(c) **Western blot analysis of phosphorylated and total insulin-like growth factor type I receptor (IGF-IR) protein expression following treatment of MCF-7, T47D and BT-474 breast cancer cells with HRGβ1 (10 ng/ml), IGF-I (10 ng/ml) or vehicle control for 5 minutes. Data are representative of three separate experiments.

Western blot analysis demonstrated that 5-minute HRGβ1 stimulation promoted EGFR, erbB3, erbB2 and EGFR phosphorylation and activation of the downstream signalling components Akt and ERK1/2 in MCF-7, T47D and BT-474 breast cancer cells, as shown in Figure 1a. Interestingly, HRGβ1 also promoted phosphorylation of IRS-1 at Y612 and at Y896 in all cell lines, but less dramatically in the BT-474 cells (Figure 1b). Treatment of HRGβ1 had no effect on either specific IGF-IR Y1316 phosphorylation or total IGF-IR protein expression levels in these cell lines, whilst, as expected, 10 ng/ml IGF-I stimulation promoted IGF-IR phosphorylation (Figure 1c).

### Insulin receptor substrate 1 associates with erbB receptors in MCF-7, T47D and BT-474 cells

**Figure 2:**
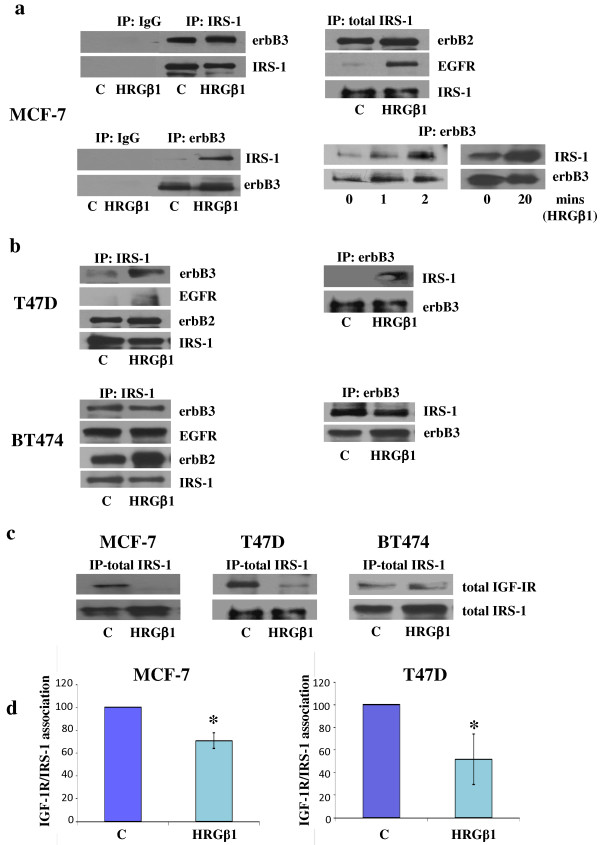
**Western blot showing erbB3, EGFR, erbB2 and insulin receptor substrate 1 protein expression**. **(a) **Immunoprecipitation (IP) with rabbit immunoglobulin G (IgG) (negative control), insulin receptor substrate 1 (IRS-1) or erbB3 antibody for MCF-7 cells treated with either heregulin β1 (HRGβ1) (10 ng/ml) or vehicle control for 5 minutes or up to 20 minutes, **(b) **immunoprecipitation with either IRS-1 or erbB3 antibody in T47D and BT-474 cells treated with either HRGβ1 (10 ng/ml) or vehicle control for 5 minutes. **(c) **Western blot analysis of IGF-IR and IRS-1 protein expression following immunoprecipitation with IRS-1 antibody in MCF-7, T47D and BT-474 cells treated with either HRGβ1 (10 ng/ml) or vehicle control for 5 minutes. **(d) **Densitometric analysis of the loss of IRS-1 association with IGF-IR following HRGβ1 treatment in MCF-7 and T47D cells. The results are expressed as means ± standard errors of the mean of at least three separate experiments. The *y*-axis represents arbitrary optical densitometric units. In MCF-7 cells * P ≤ 0.01 versus Control; In T47D cells * P ≤ 0.05 versus Control.

Immunoprecipitation and Western blot analysis were performed to examine whether IRS-1 associates with erbB receptors, notably erbB3, in the ER+ breast cancer cell lines. Western blot analysis demonstrated an interaction between erbB3 and IRS-1 under both basal and HRGβ1-primed growth conditions in MCF-7 cells (Figure 2a). The specificity of the anti-IRS-1 and anti-erbB3 antibodies used in these studies was confirmed using rabbit immunoglobulin G antibodies (Figure 2a). A HRGβ1 time course was also performed in MCF-7 cells, and maximum IRS-1 Y612 and Y896 phosphorylation levels were observed after just 2 minutes treatment time. This was sustained for up to 20 minutes (data not shown). Immunoprecipitation performed over this time span clearly showed increased association between IRS-1 and erbB3 following HRGβ1 treatment (Figure 2a). IRS-1 was also shown to associate with both EGFR and erbB2 under basal growth conditions, and these associations were enhanced following HRGβ1 treatment (Figure 2a). Similar findings were also observed for T47D cells, as shown in Figure 2b. Moreover, HRGβ1 stimulation also promoted a significant decrease in association between IRS-1 and IGF-IR in both the MCF-7 and T47D cells (Figure 2c), and densitometric analysis revealed this reduction to be statistically significant in both cell lines (*P *≤ 0.01 (*n *= 3) and *P *≤ 0.05 (*n *= 3), respectively) (Figure 2d). However, although BT-474 cells showed an association between IRS-1 and erbB3 under basal growth conditions, this was not increased further following HRGβ1 treatment (Figure 2b). In addition, there was no decrease in association between IGF-IR and IRS-1 following HRGβ1 stimulation in these cells (Figure 2c) and, as a consequence, BT-474 cells were excluded from further studies.

### erbB3 siRNA knockdown reduces heregulin β1-induced insulin receptor substrate 1 phosphorylation in MCF-7 and T47D cells

**Figure 3:**
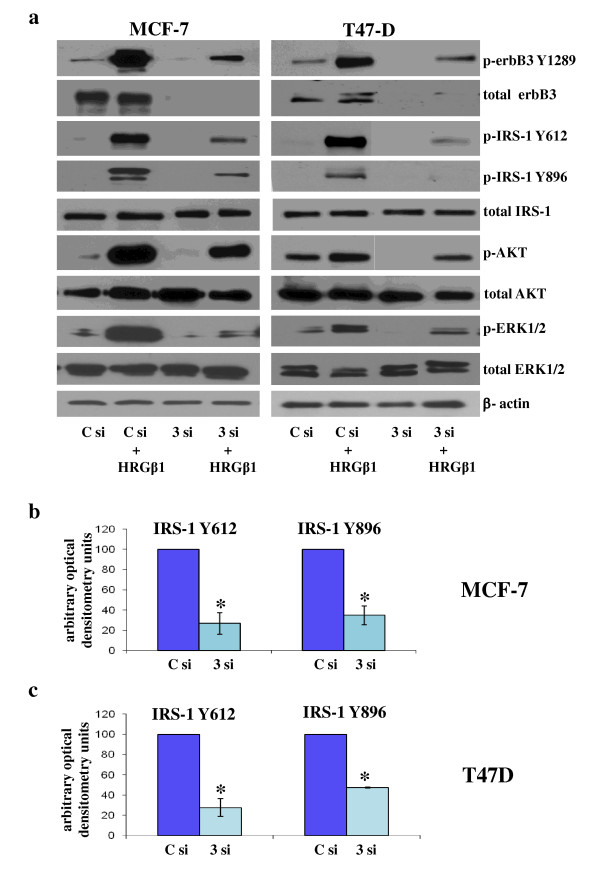
**Western blot analysis**. **(a) **Phosphorylated and total erbB3, insulin receptor substrate 1 (IRS-1), Akt, extracellular-signal regulated kinase 1/2 (ERK1/2) and β-actin protein expression in MCF-7 and T47D cells incubated in medium supplemented with **(a) **lipid and control siRNA (C si) mix (100 nM) or lipid and erbB3 siRNA (3 si) mix (100 nM) for 4 days and subsequently challenged with either heregulin β1 (HRGβ1) (10 ng/ml) or vehicle control alone for 5 minutes. Densitometric analysis of **(b) **phosphorylated IRS-1 Y612 and Y896 protein levels in MCF-7 and **(c) **T47D cells resulting from HRGβ1-primed erbB3 siRNA- versus control siRNA-treated groups. The results are expressed as means ± standard errors of the mean of at least three separate experiments. In MCF-7 cells * P ≤ 0.01 versus Control siRNA for both phosphorylation sites; In T47D cells * P ≤ 0.01 versus Control siRNA for pY612 IRS-1 and * P ≤ 0.001 versus Control siRNA for pY896 IRS-1.

Western blot analysis demonstrated that both basal and HRGβ1-primed IRS-1 Y612 and Y896 phosphorylation levels were markedly reduced following incubation of MCF-7 and T47D breast cancer cells with siRNA-targeting erbB3 protein expression. The reduction in IRS-1 Y612 and Y896 phosphorylation was found to be statistically significant following densitometric analysis (*P *≤ 0.01 (*n *= 3) and *P *≤ 0.01 (*n *= 3), respectively, for MCF-7; *P *≤ 0.01 (*n *= 3) and *P *≤ 0.001 (*n *= 3), respectively, for T47D cells) (Figures 3b and 3c). Basal and HRGβ1-primed Akt and ERK1/2 phosphorylation levels were similarly inhibited by the erbB3 siRNA treatment (Figure 3a), whilst total Akt, ERK1/2 and β-actin protein levels remained constant following total erbB3 downregulation in both cell lines.

### Insulin receptor substrate 1 siRNA knockdown reduces heregulin β1-primed erbB3 signalling

**Figure 4:**
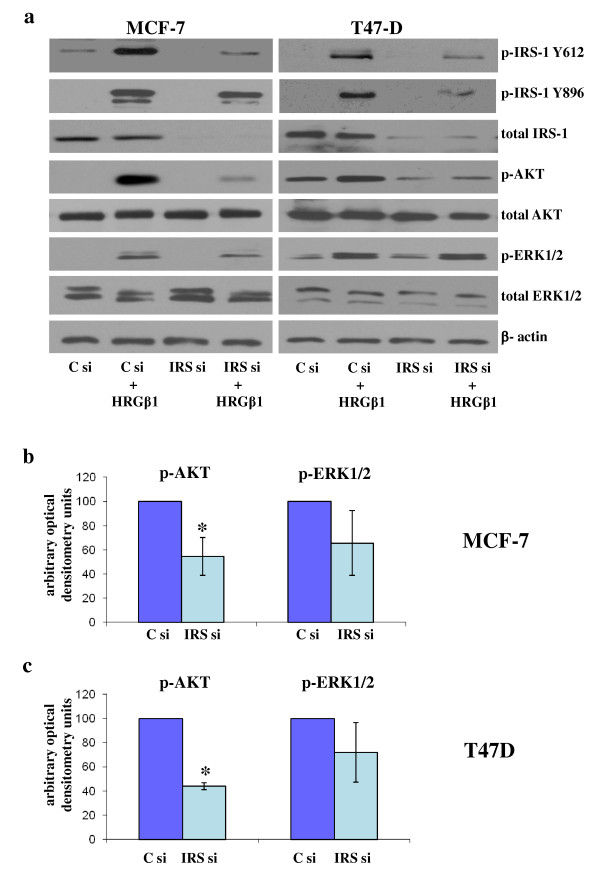
**Western blot analysis**. **(a) **Phosphorylated and total insulin receptor substrate 1 (IRS-1), Akt, extracellular-signal regulated kinase 1/2 (ERK1/2) and β-actin protein expression in MCF-7 and T47D cells incubated in medium supplemented with either lipid and control siRNA (C si) mix (100 nM) or lipid and IRS si mix (100 nM) for 4 days and subsequently challenged with either heregulin β1 (HRGβ1) (10 ng/ml) or vehicle control alone for 5 minutes. Densitometric analysis of phosphorylated Akt and ERK1/2 protein levels in **(b) **MCF-7 and **(c) **T47D cells resulting from the HRGβ1-primed IRS-1 siRNA- versus control siRNA-treated groups. The results are expressed as means ± standard errors of the mean of at least three separate experiments. In MCF-7 cells * P ≤ 0.01 versus Control siRNA; In T47D cells * P ≤ 0.001 versus Control siRNA.

Interestingly, the ability of HRGβ1 to prime Akt phosphorylation was reduced substantially following incubation of MCF-7 and T47D cells with siRNA-targeting IRS-1 protein expression as demonstrated by Western blot analysis (Figure 4a). Moreover, this decrease in Akt phosphorylation was statistically significant following densitometric analysis (*P *≤ 0.01 (*n *= 3) and *P *≤ 0.001 (*n *= 3) for MCF-7 and T47D cells, respectively) (Figures 4b and 4c). However, there was no obvious reduction in HRGβ1-induced ERK1/2 phosphorylation in these cells following IRS-1 protein downregulation. Total Akt, ERK1/2 and β-actin protein levels remained constant.

### Insulin-like growth factor type I receptor inhibition facilitates insulin receptor substrate 1 association with erbB3 and promotes heregulin β1-induced phosphorylation of insulin receptor substrate 1 Y612 in MCF-7 and T47D cells

**Figure 5:**
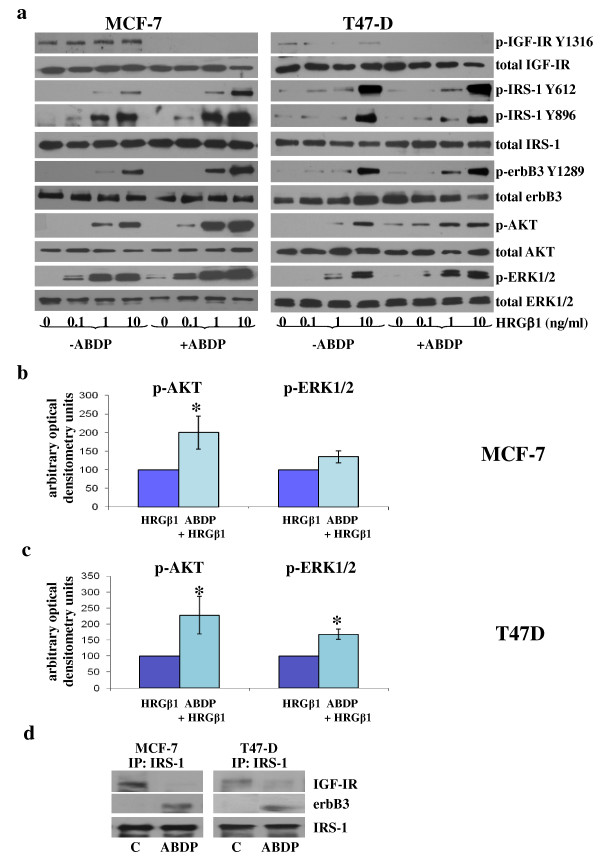
**Western blot analysis**. **(a) **Total and phosphorylated insulin-like growth factor type I receptor (IGF-IR), insulin receptor substrate 1 (IRS-1), erbB3, Akt and extracellular-signal regulated kinase 1/2 (ERK1/2) protein expression following incubation of MCF-7 and T47D cells in medium containing either the specific IGF-IR/IR tyrosine kinase inhibitor 4-anilino-5-bromo-2-[4-(2-hydroxy-3-(*N*, *N*-dimethylamino)propoxy)anilino]pyrimidine (ABDP) (1 μM) or appropriate vehicle control for 24 hours and subsequently challenged with increasing concentrations of heregulin β1 (HRGβ1) (0.1 to 10 ng/ml) or vehicle control for 5 minutes. Densitometric analysis of phosphorylated Akt and ERK1/2 protein levels in **(b) **MCF-7 and **(c) **T47D cells treated with HRGβ1 in the absence and presence of ABDP. The results are expressed as the means ± standard errors of the mean of at least three separate experiments from the 1 ng/ml HRGβ1-primed groups. **(d) **Western blot analysis of total IGF-IR, erbB3 and IRS-1 expression following immunoprecipitation (IP) with total IRS-1 antibody in MCF-7 and T47D cells incubated for 24 hours in medium containing ABDP (1 μM) or vehicle control. * P ≤ 0.05 versus HRGβ1 for both cell lines.

We next examined whether recruitment of IRS-1 by erbB3 can provide a resistance mechanism to IGF-IR-targeted therapy. A 24-hour treatment with the IGF-IR inhibitor ABDP enhanced the sensitivity of MCF-7 and T47D cells to HRGβ1, with increased phosphorylation of IRS-1 Y612, IRS-1 Y896, Akt and ERK1/2 apparent at lower concentrations of this ligand (Figure 5a). These increases were not due to increased IGF-IR activity, as specific phosphorylated IGF-IR levels were completely inhibited following ABDP treatment. Densitometric analysis showed that the increase in Akt phosphorylation was statistically significant for both MCF-7 cells (*P *≤ 0.05 (*n *= 3)) and T47D cells (*P *≤ 0.05 (*n *= 3)) at the 1 ng/ml HRGβ1 concentration (Figures 5b and 5c). The increased ERK1/2 phosphorylation observed in MCF-7 cells failed to reach statistical significance (*P *= 0.06 (*n *= 3)) (Figure 5b), although significance was reached for the T47D cells (*P *≤ 0.05 (*n *= 3)) (Figure 5c). Total protein levels remained constant following IGF-IR inhibition in both cell lines. These results were verified further in both cell lines using siRNA to specifically target IGF-IR (Additional file 2, Figure S1). Immunoprecipitation and Western blot analysis also revealed that, following treatment of MCF-7 and T47D cells with ABDP, there was a reduced association between IRS-1 and IGF-IR and an enhanced association between IRS-1 and erbB3, as shown in Figure 5d.

### siRNA knockdown of insulin receptor substrate 1 reverses the increased sensitivity to heregulin β1 observed following insulin-like growth factor type I receptor blockade in MCF-7 and T47D cells

**Figure 6:**
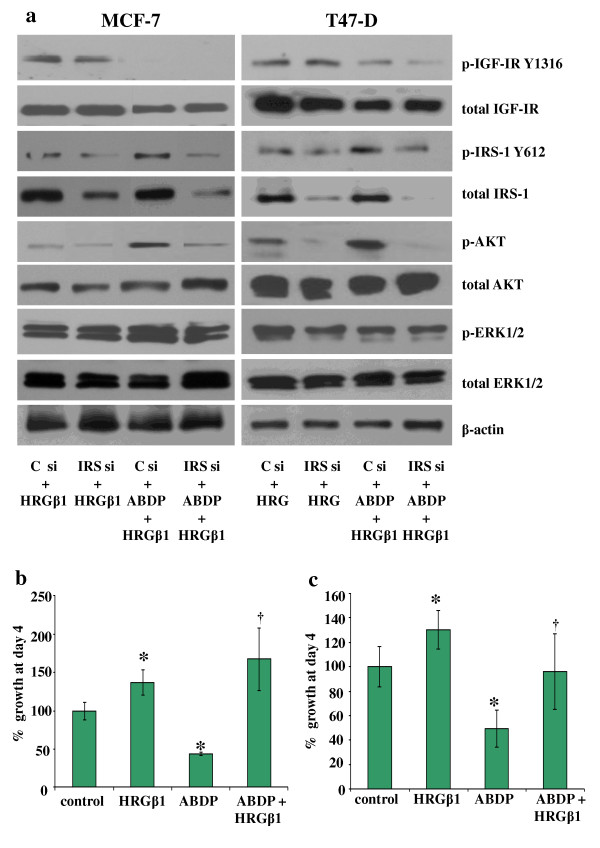
**Western blot analysis**. **(a) **Total and phosphorylated insulin-like growth factor type I receptor (IGF-IR), insulin receptor substrate 1 (IRS-1), Akt, extracellular signal-regulated kinase 1/2 (ERK1/2) and β-actin protein expression following incubation of MCF-7 and T47D cells in medium containing either lipid and control siRNA (C si) mix (100 nM) or lipid and IRS si mix (100 nM) in the absence or presence of 4-anilino-5-bromo-2-[4-(2-hydroxy-3-(*N*, *N*-dimethylamino)propoxy)anilino]pyrimidine (ABDP) (1 μM) for 2 days and subsequently challenged with heregulin β1 (HRGβ1) (10 ng/ml) for 5 minutes. Data are representative of at least three separate experiments. The effects of 4-day treatment with HRGβ1 (10 ng/ml), ABDP (0.1 μM) or a combination of HRGβ1 and ABDP on the growth of **(b) **MCF-7 cells or **(c) **T47D cells. The results are expressed as the means ± standard errors of the mean of triplicate wells and are representative of at least three separate experiments. *P ≤ 0.01 versus control, ^†^*P *≤ 0.001 versus ABDP.

The increase in HRGβ1-induced Akt phosphorylation observed following treatment of MCF-7 and T47D breast cancer cells with the IGF-IR/IR inhibitor ABDP for 24 hours was effectively blocked using siRNA specific to IRS-1 (Figure 6a). However, IRS-1 knockdown had only a small effect on HRGβ1-induced ERK1/2 phosphorylation, and it had no effect on total IGF-IR, Akt, ERK1/2 and β-actin protein levels in both these cell lines.

### Growth inhibition by ABDP can be overcome by heregulin β1 in MCF-7 and T47D cells

The growth of MCF-7 and T47D breast cancer cells was significantly increased in the presence of HRGβ1 (*P *≤ 0.001 (*n *= 3) and *P *≤ 0.01 (*n *= 3), respectively) and significantly reduced by approximately 50% in the presence of either 0.1 μM ABDP in MCF-7 cells (*P *≤ 0.001 (*n *= 3)) or 0.75 μM ABDP in T47D cells (*P *≤ 0.001 (*n *= 3)) (Figures 6b and 6c). This inhibition in cell growth observed with ABDP was potently and significantly overridden by treatment of MCF-7 and T47D cells with 10 ng/ml HRGβ1 (*P *≤ 0.001 (*n *= 3) and *P *≤ 0.01 (*n *= 3), respectively) as shown in Figures 6b and 6c.

### Phosphorylated insulin receptor substrate 1 Y612 expression positively correlates with erbB3 and insulin-like growth factor type I receptor expression in oestrogen receptor-positive clinical breast cancer material

**Figure 7:**
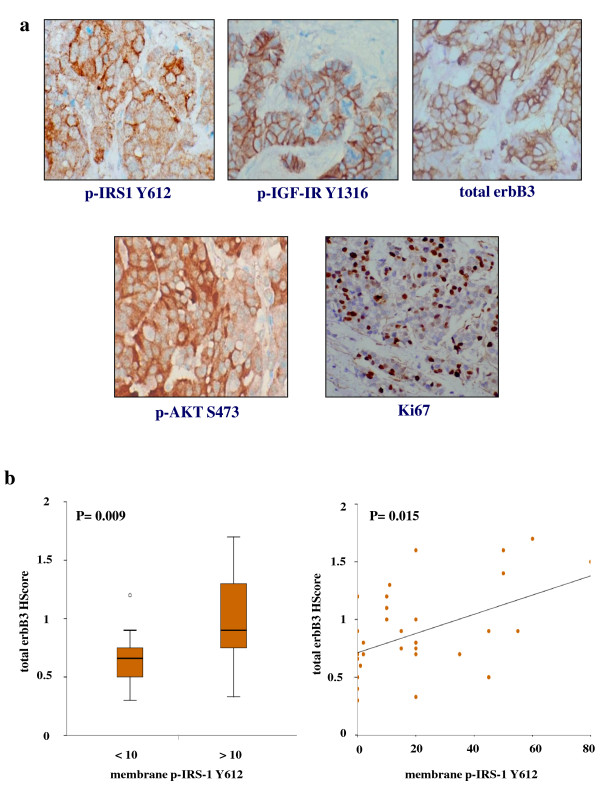
**Immunocytochemical staining**. **(a) **Phosphorylated insulin receptor substrate 1 (IRS-1) Y612, insulin-like growth factor type I receptor (IGF-IR), Y1316, total erbB3, Akt and Ki-67 in paraffin-embedded primary clinical oestrogen-positive (ER+) breast cancer sections (original magnification, ×40). **(b) **Representative boxplot and scatterplot showing statistically significant correlations between immunostaining HScore values for phosphorylated membrane IRS-1 Y612 and total membrane and cytoplasmic erbB3 in the clinical ER+ breast cancer series.

Phosphorylated IRS-1 Y612 protein was expressed in the majority of paraffin-embedded breast cancer clinical samples (range, 0 to 220; median, 61) as assessed by immunocytochemical analysis, with immunostaining localised predominantly at the plasma membrane, although some cytoplasmic staining was also observed (Figure 7a). Immunocytochemical assays for phosphorylated Akt, specific phosphorylated IGF-IR (Y1316), nuclear Ki-67 and total erbB3 were also performed as previously described on the 50 primary ER+ breast tumours [6,30,41], and the clinicopathological parameters for the clinical set of these tumours are given in Additional file 1, Table S1. A Mann-Whitney *U *test was applied to these samples to determine the relationships between phosphorylated membrane IRS-1 Y612 immunostaining (using a median HScore of 10 as a cutoff for positivity) and total membrane and cytoplasmic erbB3, membrane phosphorylated IGF-IR Y1316, membrane and cytoplasmic phosphorylated Akt and percentage of nuclear Ki-67 immunostaining HScore values. Interestingly, total membrane and cytoplasmic erbB3 expression was significantly higher in phosphorylated membrane IRS-1 Y612-positive tumours than in phosphorylated membrane IRS-1 Y612-negative tumours (*P *= 0.009 (*n *= 33)) (Figure 7b). Further analysis also revealed that phosphorylated membrane and cytoplasmic IRS-1 Y612-positive tumours (median HScore cutoff of 61) expressed higher levels of phosphorylated membrane IGF-IR Y1316 (*P *= 0.011 (*n *= 50)), phosphorylated membrane and cytoplasmic Akt (*P *≤ 0.001 (*n *= 50)) and nuclear Ki-67 (*P *= 0.022 (*n *= 40)) immunostaining than did phosphorylated membrane and cytoplasmic IRS-1 Y612-negative tumours (not shown). In addition, Spearman's rank-correlation test was applied to this group of ER+ patients. This analysis confirmed the Mann-Whitney *U *test findings revealing that phosphorylated membrane IRS-1 Y612 immunostaining positively correlated with immunostaining for total membrane and cytoplasmic erbB3 (*P *= 0.015 (*n *= 33)) (Figure 7b). Significant positive correlations between phosphorylated membrane and cytoplasmic IRS-1 Y612 and phosphorylated membrane IGF-IR (*P *= 0.025 (*n *= 50)), phosphorylated membrane and cytoplasmic Akt (*P *= 0.001 (*n *= 50)) and nuclear Ki-67 (*P *= 0.007 (*n *= 40)) were also observed (not shown).

## Discussion

IRS-1 is not restricted to binding to IR/IGF-IR but also has the capacity to interact with a variety of other proteins [21]. Recently, we reported that IRS-1 can interact with EGFR, resulting in loss of recruitment of IRS-1 by IGF-IR and reducing signalling via this receptor in an ER+, tamoxifen-resistant MCF-7 breast cancer cell line [29]. In the present study, we examined whether IRS-1 can associate with other erbB family members, notably erbB3, and whether this has a direct impact on IGF-IR signalling in three ER+ breast cancer cell lines (MCF-7, T47D and BT-474) previously shown to express IRS-1 protein [42-44].

Initial characterisation of these cell lines showed that EGFR, erbB2, erbB3 and associated downstream signalling elements MAPK and Akt were activated following HRGβ1 treatment, with this ligand having a more potent effect on phosphorylation levels in MCF-7 and T47D cells that on BT-474 cells. Interestingly, HRGβ1 treatment also increased levels of IRS-1 phosphorylation at both the Y612 and Y896 residues, with this effect being greater in MCF-7 and T47D cells than in the BT-474 cell line. The more modest effect of HRGβ1 priming of such activity in BT-474 cells most likely reflects the fact that these cells constitutively overexpress erbB2 and consequently have higher basal phosphorylation levels of all these signalling elements. As such, any increase in activity is harder to distinguish compared to the erbB2 low-expressing MCF-7 and T47D cell lines [45]. Using immunoprecipitation and Western blot analysis, we confirmed that HRGβ1-induced phosphorylation of IRS-1 was a result of IRS-1's complexing with erbB3/EGFR and erbB3/erbB2 heterodimers in both MCF-7 and T47D cells. The ability of erbB3 to heterodimerise with both EGFR and erbB2 in response to HRGβ1 stimulation explains the increased phosphorylation of IRS-1 at Y896 in these two cell lines. We have previously described the recruitment and phosphorylation of IRS-1 at this tyrosine residue by EGFR/erbB2 heterodimers in a tamoxifen-resistant MCF-7 breast cancer cell line [29]. We have previously reported that phosphorylation of IRS-1 Y612 results from recruitment and activation by IGF-IR. In the present study, however, HRGβ1-induced IRS-1 Y612 phosphorylation appeared to be IGF-IR-independent. There was no effect of this ligand on IGF-IR phosphorylation, as verified by the use of a specific pY1316 IGF-IR antibody in these cell lines [40]. Indeed, HRGβ1 treatment reduced the association of IRS-1 with IGF-IR in both cell lines. This leaves association of IRS-1 with erbB3 as the likely mediator of HRGβ1-induced IRS-1 Y612 phosphorylation in these cells.

It has previously been reported in other systems that IRS-1-erbB3 interactions can occur, as erbB3 possesses NPXY motifs within its C-terminal domain, like those observed in IGF-IR/IR, which are recognized by IRS proteins and would enable this adaptor molecule to potentially bind to this receptor [31]. Furthermore, in a study of the binding of peptides representing the physical sites of erbB3 tyrosine phosphorylation to protein microarrays comprising all Src homology 2 and phosphotyrosine binding domains encoded in the human genome, researchers predicted a potential interaction between erbB3 and IRS-1 [28]. Importantly, our studies reveal that IRS-1 has a significant functional role in erbB3 signalling in MCF-7 and T47D cells, as erbB3 knockdown using siRNA potently inhibited basal and HRGβ1-induced IRS-1, Akt and ERK1/2 phosphorylation, whilst IRS-1 siRNA similarly reduced HRGβ1-induced Akt and, to a modest degree, ERK1/2 activity in these cells. As ERK1/2 activity was not significantly altered following IRS-1 knockdown, this would suggest that an IRS-1-independent mechanism underlying HRGβ1-induced ERK1/2 activity was at work in our cell lines. Consequently, the remainder of our study focused primarily on IRS-1 Y612/Akt phosphorylation, as this appeared to be the IRS-1-dependent pathway in response to HRGβ1 in our cell models. In BT-474 cells, there was a strong basal association between IRS-1 and erbB3, as observed in immunoprecipitation studies, which could not be enhanced further by exogenous ligand stimulation. Again, this could be due to the high constitutive erbB2 activity present within these cells masking the exogenous stimulatory effects of HRGβ1 treatment. Moreover, IRS-1 itself may potentially be more freely available to interact with erbB3 in these cells, as they have somewhat less IGF-IR protein with which to associate compared to MCF-7 and T47D cells, as shown in this study and as shown elsewhere previously [46]. As this HRGβ1-induced association between IRS-1 and erbB3 was not evident in the BT-474 cells, these cells were omitted from further study.

As mentioned previously, another interesting phenomenon noted in these studies is the finding that whilst HRGβ1 treatment enhanced erbB3-IRS-1 interactions, it also promoted a decrease in the association between IRS-1 and IGF-IR, an effect that was clearly apparent in the MCF-7 and T47D cell lines. This finding suggests that the enhanced physical interaction between erbB3 and IRS-1 following HRGβ1 treatment may serve to limit the availability of IRS-1 to associate with IGF-IR, potentially resulting in inhibition of signalling via this receptor. Indeed, as mentioned above, we have previously reported that EGFR can similarly suppress IGF-IR signalling through such a mechanism in a tamoxifen-resistant MCF-7 cell line [29]. A potential consequence of the ability of HRGβ1/erbB3 signalling to suppress IGF-IR signalling activity is that such a mechanism could severely affect the efficacy of IGF-IR-targeted agents in these breast cancer cells. Indeed, there is now evidence emerging from experimental breast cancer cell models implicating a role for erbB receptors in resistance to IGF-IR blockade, with Haluska and colleagues [47] showing that EGFR/erbB2 signalling can confer resistance to the IGF-IR tyrosine kinase inhibitor BMS-536924 in MCF-7 cells. In our present study, a role for erbB3 signalling in resistance to IGF-IR blockade is also clearly implicated, as HRGβ1 readily overcame the growth-inhibitory effects of the IGF-IR/IR tyrosine kinase inhibitor ABDP in the MCF-7 and T47D cell lines. ABDP is a novel dual IGF-IR/IR tyrosine kinase inhibitor that has previously been reported to potently inhibit IGF-IR signalling in breast and prostate cancer cell lines [40]. Blockade of IGF-IR signalling in these cells using ABDP also enhanced responses to HRGβ1 in both cell lines, with phosphorylation of IRS-1 Y612, Akt and ERK1/2 apparent at lower concentrations of this ligand and with a greater magnitude of phosphorylation also observed at the highest concentrations of HRGβ1 in ABDP-treated compared to untreated cells. Similar results were observed when IGF-IR signalling was blocked using an IGF-IR siRNA. This rapid enhancement of HRGβ1 signalling by IGF-IR inhibition is likely a consequence of two mechanisms. The first is an IRS-1-mediated mechanism, which immunoprecipitation and Western blot analysis revealed a loss of IRS-1 association with IGF-IR and an increased association of this adaptor protein with erbB3 in MCF-7 and T47D cells following treatment with ABDP, mirroring results observed for HRGβ1 treatment alone in these cell types. Thus, HRGβ1 signalling was enhanced as a result of increased availability and association of IRS-1 with erbB3. This is further supported by the finding that knockdown of IRS-1 protein levels by siRNA not only reduced HRGβ1-primed Akt phosphorylation but also prevented the ABDP-induced sensitisation of the cells to this ligand, greatly reducing signalling via Akt in particular. The second is that an erbB3-dependent mechanism appears to play a role, as IGF-IR inhibition by either ABDP or IGF-IR siRNA knockdown also enhanced HRGβ1-induced erbB3 phosphorylation, with this effect being most apparent in the MCF-7 cells. The reasons behind this effect remain unclear, although similar findings have been reported in hepatocellular carcinoma cells treated with the novel IGF-IR monoclonal antibody AVE1642 [39]. One possible mechanism was recently identified by Gijsen and colleagues [48], who reported that blockade of Akt can activate ADAM17 (ADAM metallopeptidase domain 17) in erbB2-overexpressing breast cancer cells, leading to release of heregulins, which can act in an autocrine manner to activate erbB3. As IGF-IR blockade can acutely inhibit Akt activity in our cell lines, such a mechanism may explain the subsequent phosphorylation of erbB3; however, further studies are required to confirm this hypothesis. Interestingly, IRS-1 knockdown was not as effective in reducing HRGβ1-induced ERK1/2 activity compared to Akt activity in ABDP-treated MCF-7 and T47D cell lines. One possible explanation for this is that the increased erbB3 phosphorylation observed in response to ABDP may provide the input maintaining ERK1/2 phosphorylation in these cells; however, further investigation into this mechanism is required and is currently ongoing.

To determine whether this novel association between IRS-1 and erbB3 identified in our ER+ cell lines could also have clinical relevance, an exploratory study was performed in a small series of ER+ clinical breast tumours. An immunocytochemical assay was developed to detect phosphorylated IRS-1 Y612 and associations with erbB3, and other clinical markers were assessed. Importantly, a significant positive correlation between IRS-1 Y612 phosphorylation and total erbB3 expression was observed in these ER+ primary breast tumours. As the majority of these ER+ tumours were found to express low and/or negative erbB2 levels, these findings directly support our cell line work and suggest that an association between erbB3 and IRS-1 may well occur within ER+ breast tumours. The link between IRS-1 Y612 phosphorylation levels and Akt activity identified in the cell lines was also observed in the clinical samples, with significant correlations between phosphorylated levels of IRS-1 Y612 and Akt in ER+ patients. Moreover, there was a significant correlation between IRS-1 Y612 and the proliferation marker Ki-67 in these ER+ tumours, suggesting that the potential interplay between erbB3, IRS-1 and Akt in these tumours may culminate in driving cell proliferation. However, for such signalling to arise, heregulins must be synthesized and accessible within the cancer milieu. Importantly, our previous findings based on the same clinical breast cancer series used in this study, as well as others [30,49], clearly demonstrate that neuregulins such as HRGβ1 are ubiquitously expressed in clinical breast tissue, thus making such interplay a distinct possibility and warranting a more extensive study to be carried out in a larger breast cancer series. In light of these findings, the recent suggestion that IRS-1 should be considered as a biomarker for IGF-IR activity in cancers susceptible to IGF-IR targeting [50] should be viewed with a degree of caution, especially in cancer types that also express erbB receptors and their ligands.

## Conclusions

These and previous findings identify IRS-1 as a key signalling component for both IGF-IR and erbB receptor tyrosine kinases in ER+ breast cancer cells and as an important convergence point for cross-talk between these two receptor tyrosine kinase families. These studies provide further evidence that this versatile adaptor molecule may provide an adaptive resistance mechanism when either of these receptor families is targeted individually. Consequently, targeting IRS-1 alongside such agents may prove to be a more effective strategy for the treatment of ER+ breast cancer, particularly when heregulins are abundant. Although direct targeting of IRS-1 may prove to be problematic, it may be achievable in ER+ breast cancer with the use of antihormones, as IRS-1 is an oestrogen-regulated gene [8]. Indeed, recent reports have provided evidence that such a therapeutic strategy may prove highly effective with the IGF-IR inhibitor NVP-AEW541 when used in combination with an aromatase inhibitor by synergistically inducing apoptosis in aromatase-expressing MCF-7 and T47D cells *in vitro *[51] and with a novel anti-IGF-IR antibody when combined with tamoxifen-suppressing breast tumour cell growth *in vivo *[52]. However, it should be noted that it remains to be determined whether reduced IRS-1 expression is a major contributing factor to the improved response of the combination treatments utilised in these studies.

## Abbreviations

ABDP: 4-anilino-5-bromo-2-[4-(2-hydroxy-3-(*N*, *N*-dimethylamino)propoxy)anilino]pyrimidine; BSA: bovine serum albumin; EGFR: epidermal growth factor receptor; ER: oestrogen receptor; ERK1/2: extracellular signal-regulated kinase 1/2; FCS: foetal calf serum; HRGβ1: heregulin β1; IGF-IR: insulin-like growth factor type I receptor; IRS-1: insulin receptor substrate 1; MAPK: mitogen-activated protein kinase; PBS: phosphate-buffered saline; PI3K: phosphatidylinositol 3-kinase; RPMI: phenol red-free Roswell Park Memorial Institute; siRNA: small interfering RNA; TBS: Tris-buffered saline; Y: tyrosine.

## Competing interests

IRH, JMWG and RIN are in receipt of funding from AstraZeneca, and JFR is in receipt of funding from Amgen. RIN is also a member of an advisory board for AstraZeneca. All other authors have no conflicts of interest.

## Authors' contributions

IRH conceived of the study, participated in its design and execution and helped draft the manuscript. JMK drafted the manuscript, carried out all the Western blot analyses and conducted all siRNA and cell culture studies, including growth studies with the help and support of DB. JMWG carried out the immunocytochemistry on a small series of 50 primary breast tumours excised from ER+ patients recruited by JFR and IOE. RIN participated in the design and coordination of the study and helped to draft the manuscript. All authors read and approved the final manuscript.

## Supplementary Material

Additional file 1 **Clinicopathological parameters for oestrogen receptor-positive breast tumour set**. Table S1 gives the clinicopathological parameters of a small historical series of 50 primary tumours excised from oestrogen receptor-positive (ER+) patients with histologically proven breast cancer who presented for surgery at the Nottingham City Hospital. No patient had previously received any form of adjuvant endocrinological or cytotoxic therapy. EGFR = epidermal growth factor receptor. Click here for file

Additional file 2 **Effect of insulin-like growth factor receptor knockdown on heregulin β1 signalling in MCF-7 and T47D cells**. Figure S1 shows the results of Western blot analysis of total insulin-like growth factor type I receptor (IGF-IR), phosphorylated and total insulin receptor substrate 1 (IRS-1), erbB3, Akt, ERK1/2 and β-actin protein expression in MCF-7 and T47D cells following incubation with either lipid and C si mix (100 nM) or lipid and IGF-IR siRNA (IGF si) mix (100 nM) for 4 days and subsequently challenged with either heregulin β1 (HRGβ1) (10 ng/ml) or vehicle control alone for 5 minutes. Data are representative of three separate experiments. p-AKT = phosphorylated Akt; pERK1/2 = phosphorylated extracellular signal-regulated kinase 1/2; c Si = siRNA control pool. Click here for file

## References

[B1] Sachdev D Yee D The IGF system and breast cancer Endocr Rel Cancer 2001 8 197 209 10.1677/erc.0.0080197 11566611

[B2] Surmacz E Function of the IGF-I receptor in breast cancer J Mammary Gland Biol Neoplasia 2000 5 95 105 10.1023/A:1009523501499 10791772

[B3] Fagan DH Yee D Crosstalk between IGF1R and estrogen receptor signaling in breast cancer J Mammary Gland Biol Neoplasia 2008 13 423 429 10.1007/s10911-008-9098-0 19003523 19003523

[B4] Surmacz E Guvakova MA Nolan MK Nicosia RF Sciacca L Type I insulin-like growth factor receptor function in breast cancer Breast Cancer Res Treat 1998 47 255 267 10.1023/A:1005907101686 9516080

[B5] Happerfield LC Miles DW Barnes DM Thomsen LL Smith P Hanby A The localization of the insulin-like growth factor receptor 1 (IGFR-1) in benign and malignant breast tissue J Pathol 1997 183 412 417 10.1002/(SICI)1096-9896(199712)183:4<412::AID-PATH944>3.0.CO;2-4 9496257

[B6] Gee JM Robertson JF Gutteridge E Ellis IO Pinder SE Rubini M Nicholson RI Epidermal growth factor receptor/HER2/insulin-like growth factor receptor signalling and oestrogen receptor activity in clinical breast cancer Endocr Relat Cancer 2005 12 Suppl 1 S99 S111 16113104 10.1677/erc.1.01005

[B7] Stewart AJ Johnson MD May FE Westley BR Role of insulin-like growth factors and the type I insulin-like growth factor receptor in the estrogen-stimulated proliferation of human breast cancer cells J Biol Chem 1990 265 21172 21178 2174437

[B8] Lee AV Jackson JG Gooch JL Hilsenbeck SG Coronado-Heinsohn E Osborne CK Yee D Enhancement of insulin-like growth factor signaling in human breast cancer: estrogen regulation of insulin receptor substrate-1 expression *in vitro *and *in vivo* Mol Endocrinol 1999 13 787 796 10.1210/me.13.5.787 10319328

[B9] Hamelers IH Steenbergh PH Interactions between estrogen and insulin-like growth factor signaling pathways in human breast tumor cells Endocr Relat Cancer 2003 10 331 345 10.1677/erc.0.0100331 12790794

[B10] Yee D Lee AV Crosstalk between the insulin-like growth factors and estrogens in breast cancer J Mammary Gland Biol Neoplasia 2000 5 107 115 10.1023/A:1009575518338 10791773

[B11] Rocha RL Hilsenbeck SG Jackson JG VanDenBerg CL Weng C Lee AV Yee D Insulin-like growth factor binding protein-3 and insulin receptor substrate-1 in breast cancer: correlation with clinical parameters and disease-free survival Clin Cancer Res 1997 3 103 109 9815544

[B12] Turner BC Haffty BG Narayanan L Yuan J Havre PA Gumbs AA Kaplan L Burgaud JL Carter D Baserga R Glazer PM Insulin-like growth factor-I receptor overexpression mediates cellular radioresistance and local breast cancer recurrence after lumpectomy and radiation Cancer Res 1997 57 3079 3083 9242428

[B13] Gualberto A Pollak M Emerging role of insulin-like growth factor receptor inhibitors in oncology: early clinical trial results and future directions Oncogene 2009 28 3009 3021 10.1038/onc.2009.172 19581933

[B14] Lee YH White MF Insulin receptor substrate proteins and diabetes Arch Pharm Res 2004 27 361 370 10.1007/BF02980074 15180298

[B15] White MF The insulin signalling system and the IRS proteins Diabetologia 1997 40 Suppl 2 S2 S17 9248696 10.1007/s001250051387

[B16] Lawlor MA Alessi DR PKB/Akt: a key mediator of cell proliferation, survival and insulin responses? J Cell Sci 2001 114 2903 2910 11686294 11686294 10.1242/jcs.114.16.2903

[B17] Nicholson KM Anderson NG The protein kinase B/Akt signalling pathway in human malignancy Cell Signal 2002 14 381 395 10.1016/S0898-6568(01)00271-6 11882383

[B18] Chang Q Li Y White MF Fletcher JA Xiao S Constitutive activation of insulin receptor substrate 1 is a frequent event in human tumors: therapeutic implications Cancer Res 2002 62 6035 6038 12414625

[B19] Dearth RK Cui X Kim HJ Kuiatse I Lawrence NA Zhang X Divisova J Britton OL Mohsin S Allred DC Hadsell DL Lee AV Mammary tumorigenesis and metastasis caused by overexpression of insulin receptor substrate (IRS)-1 or IRS-2 Mol Cell Biol 2006 26 9302 9314 10.1128/MCB.00260-06 17030631 PMC1698542

[B20] Koda M Sulkowska M Kanczuga-Koda L Golaszewska J Kisielewski W Baltaziak M Wincewicz A Sulkowski S Expression of the insulin receptor substrate 1 in primary tumors and lymph node metastases in breast cancer: correlations with Bcl-xL and Bax proteins Neoplasma 2005 52 361 363 16151579

[B21] Dearth RK Cui X Kim HJ Hadsell DL Lee AV Oncogenic transformation by the signaling adaptor proteins insulin receptor substrate (IRS)-1 and IRS-2 Cell Cycle 2007 6 705 713 10.4161/cc.6.6.4035 17374994

[B22] Nicholson RI McClelland RA Gee JMW Manning DL Cannon P Robertson JF Ellis IO Blamey RW Epidermal growth factor receptor expression in breast cancer: association with response to endocrine therapy Breast Cancer Res Treat 1994 29 117 125 10.1007/BF00666187 7912565

[B23] Knowlden JM Hutcheson IR Jones HE Madden T Gee JM Harper ME Barrow D Wakeling AE Nicholson RI Elevated levels of epidermal growth factor receptor/c-erbB2 heterodimers mediate an autocrine growth regulatory pathway in tamoxifen-resistant MCF-7 cells Endocrinology 2003 144 1032 1044 10.1210/en.2002-220620 12586780

[B24] Hiscox S Morgan L Barrow D Dutkowskil C Wakeling A Nicholson RI Tamoxifen resistance in breast cancer cells is accompanied by an enhanced motile and invasive phenotype: inhibition by gefitinib ('Iressa', ZD1839) Clin Exp Metastasis 2004 21 201 212 15387370 10.1023/b:clin.0000037697.76011.1d

[B25] Nicholson RI Hutcheson IR Hiscox SE Knowlden JM Giles M Barrow D Gee JM Growth factor signalling and resistance to selective oestrogen receptor modulators and pure anti-oestrogens: the use of anti-growth factor therapies to treat or delay endocrine resistance in breast cancer Endocr Relat Cancer 2005 12 Suppl 1 S29 S36 16113097 10.1677/erc.1.00991

[B26] Fujioka T Kim JH Adachi H Saito K Tsujimoto M Yokoyama S Ui M Further evidence for the involvement of insulin receptor substrates in epidermal growth factor-induced activation of phosphatidylinositol 3-kinase Eur J Biochem 2001 268 4158 4168 10.1046/j.1432-1327.2001.02327.x 11488908

[B27] Fujioka T Ui M Involvement of insulin receptor substrates in epidermal growth factor induced activation of phosphatidylinositol 3-kinase in rat hepatocyte primary culture Eur J Biochem 2001 268 25 34 10.1046/j.1432-1327.2001.01831.x 11121098

[B28] Jones RB Gordus A Krall JA MacBeath G A quantitative protein interaction network for the ErbB receptors using protein microarrays Nature 2006 439 168 174 10.1038/nature04177 16273093

[B29] Knowlden JM Jones HE Barrow D Gee JM Nicholson RI Hutcheson IR Insulin receptor substrate-1 involvement in epidermal growth factor receptor and insulin-like growth factor receptor signalling: implication for gefitinib ('Iressa') response and resistance Breast Cancer Res Treat 2007 111 79 91 17902048 10.1007/s10549-007-9763-9

[B30] Hutcheson IR Knowlden JM Hiscox SE Barrow D Gee JM Robertson JF Ellis IO Nicholson RI Heregulin β1 drives gefitinib-resistant growth and invasion in tamoxifen-resistant MCF-7 breast cancer cells Breast Cancer Res 2007 9 R50 10.1186/bcr1754 17686159 PMC2206726

[B31] Prigent SA Gullick WJ Identification of c-erbB-3 binding sites for phosphatidylinositol 3'-kinase and SHC using an EGF receptor/c-erbB-3 chimera EMBO J 1994 13 2831 2841 8026468 10.1002/j.1460-2075.1994.tb06577.x PMC395164

[B32] Baselga J Swain SM Novel anticancer targets: revisiting ERBB2 and discovering ERBB3 Nat Rev Cancer 2009 9 463 475 10.1038/nrc2656 19536107

[B33] Hamburger AW The role of ErbB3 and its binding partners in breast cancer progression and resistance to hormone and tyrosine kinase directed therapies J Mammary Gland Biol Neoplasia 2008 13 225 233 10.1007/s10911-008-9077-5 18425425 PMC3709461

[B34] Lemoine NR Barnes DM Hollywood DP Hughes CM Smith P Dublin E Prigent SA Gullick WJ Hurst HC Expression of the ERBB3 gene product in breast cancer Br J Cancer 1992 66 1116 1121 10.1038/bjc.1992.420 1333787 PMC1978009

[B35] Naidu R Yadav M Nair S Kutty MK Expression of c-erbB3 protein in primary breast carcinomas Br J Cancer 1998 78 1385 1390 10.1038/bjc.1998.689 9823984 PMC2063171

[B36] Travis A Pinder SE Robertson JF Bell JA Wencyk P Gullick WJ Nicholson RI Poller DN Blamey RW Elston CW Ellis IO C-erbB3 in human breast carcinoma: expression and relation to prognosis and established prognostic indicators Cancer 1996 74 229 233 10.1038/bjc.1996.342 PMC2074568 8688326

[B37] Pinkas-Kramarski R Soussan L Waterman H Levkowitz G Alroy I Klapper L Lavi S Seger R Ratzkin BJ Sela M Yarden Y Diversification of Neu differentiation factor and epidermal growth factor signaling by combinatorial receptor interactions EMBO J 1996 15 2452 2467 8665853 PMC450177

[B38] Wiseman SM Makretsov N Nielsen TO Gilks B Yorida E Cheang M Turbin D Gelmon K Huntsman DG Coexpression of the type 1 growth factor receptor family members HER-1, HER-2, and HER-3 has a synergistic negative prognostic effect on breast carcinoma survival Cancer 2005 103 1770 1777 10.1002/cncr.20970 15770691

[B39] Desbois-Mouthon C Baron A Blivet-Van Eggelpoël MJ Fartoux L Venot C Bladt F Housset C Rosmorduc O Insulin-like growth factor-1 receptor inhibition induces a resistance mechanism via the epidermal growth factor receptor/HER3/AKT signaling pathway: rational basis for cotargeting insulin-like growth factor-1 receptor and epidermal growth factor receptor in hepatocellular carcinoma Clin Cancer Res 2009 15 5445 5456 10.1158/1078-0432.CCR-08-2980 19706799

[B40] Jones HE Gee JMW Barrow D Tonge D Holloway B Nicholson RI Inhibition of insulin receptor isoform-A signalling restores sensitivity to gefitinib in previously *de novo *resistant colon cancer cells Br J Cancer 2006 95 172 180 10.1038/sj.bjc.6603237 16819546 16819546 PMC2360620

[B41] Knowlden JM Gee JM Seery LT Farrow L Gullick WJ Ellis IO Blamey RW Robertson JF Nicholson R c-erbB3 and c-erbB4 expression is a feature of the endocrine responsive phenotype in clinical breast cancer Oncogene 1998 17 1949 1957 10.1038/sj.onc.1202107 9788438

[B42] Byron SA Horwitz KB Richer JK Lange CA Zhang X Yee D Insulin receptor substrates mediate distinct biological responses to insulin-like growth factor receptor activation in breast cancer cells Br J Cancer 2006 95 1220 1228 10.1038/sj.bjc.6603354 17043687 PMC2360584

[B43] Mukohara T Shimada H Ogasawara N Wanikawa R Shimomura M Nakatsura T Ishii G Park JO Jänne PA Saijo N Minami H Sensitivity of breast cancer cell lines to the novel insulin-like growth factor-1 receptor (IGF-IR) inhibitor NVP-AEW541 is dependent on the level of IRS-1 expression Cancer Lett 2009 282 14 24 10.1016/j.canlet.2009.02.056 19345478

[B44] Jackson JG White MF Yee D Insulin receptor substrate-1 is the predominant signaling molecule activated by insulin-like growth factor-I, insulin, and interleukin-4 in estrogen receptor-positive human breast cancer cells J Biol Chem 1998 273 9994 10003 10.1074/jbc.273.16.9994 9545345

[B45] Alimandi M Romano A Curia MC Muraro R Fedi P Aaronson SA Di Fiore PP Kraus MH Cooperative signaling of ErbB3 and ErbB2 in neoplastic transformation and human mammary carcinomas Oncogene 1995 10 1813 1821 7538656

[B46] Chakraborty AK Liang K DiGiovanna MP Co-targeting insulin-like growth factor I receptor and HER2: dramatic effects of HER2 inhibitors on nonoverexpressing breast cancer Cancer Res 2008 68 1538 1545 10.1158/0008-5472.CAN-07-5935 18316619

[B47] Haluska P Carboni JM Ten Eyck C Attar RM Hou X Yu C Sagar M Wong TW Gottardis MM Erlichman CM HER receptor signaling confers resistance to the insulin-like growth factor-I receptor inhibitor, BMS-536924 Mol Cancer Ther 2008 7 2589 2598 10.1158/1535-7163.MCT-08-0493 18765823 PMC2614316

[B48] Gijsen M King P Perera T Parker PJ Harris AL Larijani B Kong A HER2 phosphorylation is maintained by a PKB negative feedback loop in response to anti-HER2 herceptin in breast cancer PLoS Biol 2010 8 e1000563 10.1371/journal.pbio.1000563 21203579 PMC3006345

[B49] Dunn M Sinha P Campbell R Blackburn E Levinson N Rampaul R Bates T Humphreys S Gullick WJ Co-expression of neuregulins 1, 2, 3 and 4 in human breast cancer J Pathol 2004 203 672 680 10.1002/path.1561 15141382

[B50] Baserga R The insulin receptor substrate-1: a biomarker for cancer? Exp Cell Res 2009 315 727 732 10.1016/j.yexcr.2008.09.017 18851963

[B51] Lisztwan J Pornon A Chen B Chen S Evans DB The aromatase inhibitor letrozole and inhibitors of insulin-like growth factor I receptor synergistically induce apoptosis in *in vitro *models of estrogen-dependent breast cancer Breast Cancer Res 2008 10 R56 10.1186/bcr2113 18611244 PMC2575527

[B52] Ye JJ Liang SJ Guo N Li SL Wu AM Giannini S Sachdev D Yee D Brünner N Ikle D Fujita-Yamaguchi Y Combined effects of tamoxifen and a chimeric humanized single chain antibody against the type I IGF receptor on breast tumor growth *in vivo* Horm Metab Res 2003 35 836 842 14710366 10.1055/s-2004-814145

