# Supplementary material for: erbB3 recruitment of insulin receptor substrate 1 modulates insulin-like growth factor receptor signalling in oestrogen receptor-positive breast cancer cell lines
Source: Breast Cancer Res. 2011 Sep 22;13(5):R93. doi: 10.1186/bcr3018 (PMC3262205; doi:10.1186/bcr3018)
Supplement: Additional file 1 — Clinicopathological parameters for oestrogen receptor-positive breast tumour set. Table S1 gives the clinicopathological parameters of a small historical series of 50 primary tumours excised from oestrogen receptor-positive (ER+) patients with histologically proven breast cancer who presented for surgery at the Nottingham City Hospital. No patient had previously received any form of adjuvant endocrinological or cytotoxic therapy. EGFR = epidermal growth factor receptor. [file bcr3018-S1.PDF]

Table S1. Clinical-pathological parameters for ER+ breast tumour set.

	No.	%
**Tumour grade**		
1	5	10.2
2	27	55.1
3	17	34.7
**EGFR (membrane) status**		
+ve	11	27.5
-ve	29	72.5
**erbB2 (membrane) status**		
+ve	10	25
-ve	30	75
**Menopausal status**		
Premenopausal	16	32
Postmenopausal	34	68
**Site of disease**		
Locally advanced	12	35.3
Metastatic	22	64.7
**Ki-67 index**		
<10	7	17.5
10-30	33	82.5
