# Supplementary figures and images for: erbB3 recruitment of insulin receptor substrate 1 modulates insulin-like growth factor receptor signalling in oestrogen receptor-positive breast cancer cell lines
Source: Breast Cancer Res. 2011 Sep 22;13(5):R93. doi: 10.1186/bcr3018 (PMC3262205; doi:10.1186/bcr3018)

**Figure S1**

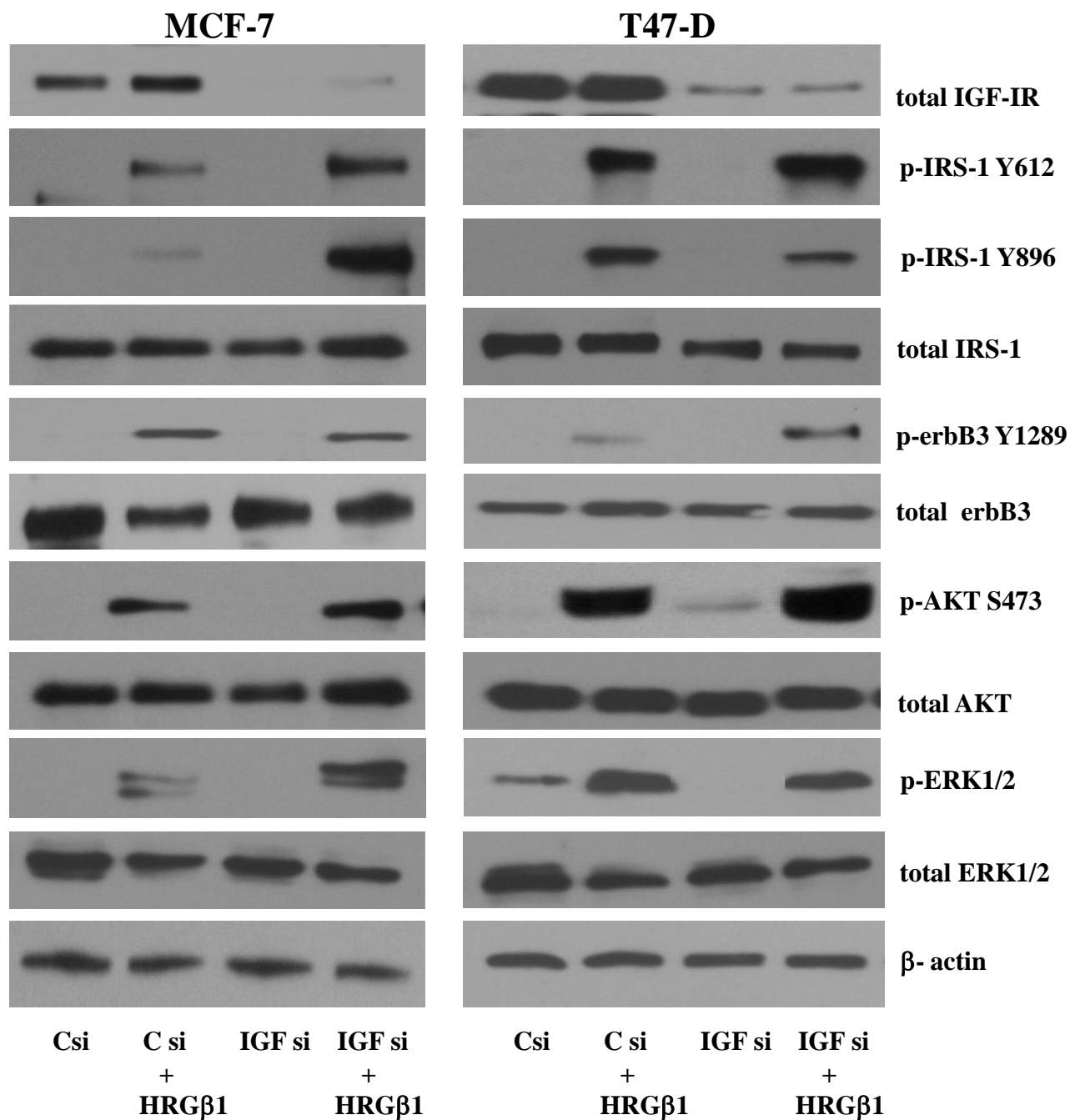

Supplement: Additional file 2 — Effect of insulin-like growth factor receptor knockdown on heregulin β1 signalling in MCF-7 and T47D cells. Figure S1 shows the results of Western blot analysis of total insulin-like growth factor type I receptor (IGF-IR), phosphorylated and total insulin receptor substrate 1 (IRS-1), erbB3, Akt, ERK1/2 and β-actin protein expression in MCF-7 and T47D cells following incubation with either lipid and C si mix (100 nM) or lipid and IGF-IR siRNA (IGF si) mix (100 nM) for 4 days and subsequently challenged with either heregulin β1 (HRGβ1) (10 ng/ml) or vehicle control alone for 5 minutes. Data are representative of three separate experiments. p-AKT = phosphorylated Akt; pERK1/2 = phosphorylated extracellular signal-regulated kinase 1/2; c Si = siRNA control pool. [file bcr3018-S2.PDF]
